# Preinjury, injury and post-injury factors leading to death in children and young people who were victims of knife crime in England between 2019 and 2024: a review of the National Child Mortality Database

**DOI:** 10.1136/emermed-2025-215154

**Published:** 2026-04-20

**Authors:** Tom Roberts, David Odd, John Coveney, Stacey Webster, Jade Levell, Sylvia Stoianova, Vicky Sleap, Tom Williams, Robin Marlow, Karen Luyt, Edward Carlton

**Affiliations:** 1 Emergency Department, North Bristol NHS Trust, Westbury on Trym, UK; 2 Translational Health Sciences, University of Bristol Medical School, Bristol, UK; 3 National Child Mortality Database, Bristol Medical School, University of Bristol, Bristol, UK; 4 Neonatology, NHS North Bristol NHS Trust, Bristol, UK; 5 Emergency Department, Bristol Royal Hospital for Children, Bristol, UK; 6 Academic Department of Military Emergency Medicine, Royal Centre for Defence Medicine, Birmingham, UK; 7 University of Bristol School for Policy Studies, Bristol, UK; 8 Paediatric Emergency Department, Bristol Royal Hospital for Children, Bristol, UK; 9 University of Bristol Population Health Sciences, Bristol, UK; 10 The National Institute for Health and Care Research Applied Research Collaboration West, University Hospitals Bristol and Weston NHS Foundation Trust, Bristol, UK

**Keywords:** violence, Mortality

## Abstract

**Background:**

Knife-related deaths in children present a serious public health challenge. This study reports the demographics and preinjury, injury and postinjury factors associated with death in children and young people (CYP), under the age of 18 years, who died of knife wounds in England between 2019 and 2024.

**Methods:**

A retrospective cohort review of the English National Child Mortality Database between April 2019 and March 2024. Rates of death were corrected for population size using the 2021 census. Preinjury, injury and postinjury factors associated with death are reported descriptively.

**Results:**

145 CYP died of knife wounds. The mean age was 14.4 years (SD 4.2) and 90.3% (n=131) were male. The rates of death per 100 000 CYP, per year were highest in children of ‘Black/Black British’ ethnicity (1.40 (95% CI 1.03 to 1.86)), with an incidence rate ratio (IRR) of 13.29 (95% CI 8.23 to 20.00), compared with CYP of ‘White’ ethnicity. Children living in the most deprived areas, had a greater than seven times higher risk of death (IRR 7.48 (95% CI 3.22 to 17.29), compared to CYP living in the least deprived areas. Of the 57 cases available for detailed analysis, injuries to the chest and neck were responsible for the fatal injuries in 75.9% of cases (n=44) and 60.3% (n=35) died before reaching hospital. A thoracotomy was performed in 56.9% (n=33) of cases. Prior to death, 75.4% (n=43) had been known to social services and 57.9% (n=33) had experienced domestic violence and abuse. Neurodiversity or mental health concerns were reported in 50.9% (n=29) of CYP.

**Conclusions:**

Death of CYP secondary to knife wounds occurred in all regions of England. Many children are exposed to adverse childhood experiences before death and known to statutory services. The identification of interventions to decrease the risk to children from knife violence remains a priority.

**WHAT IS ALREADY KNOWN ON THIS TOPIC**

- While knife - related deaths remain a significant public health challenge in England, the demographics, injury patterns and medical interventions provided to those fatally injured have not been previously described in detail.

**WHAT THIS STUDY ADDS**

- Most children who were victims of stabbing deaths were involved with statutory services prior to injury, with possible intervenable points prior to death. Children and young people (CYP) of ‘Black/Black British’ ethnicity and those living in the most deprived areas were at the highest risk of death. CYP often presented with multiple knife wounds, and thoracic injuries were most frequently associated with death.

**HOW THIS STUDY MIGHT AFFECT RESEARCH, PRACTICE OR POLICY**

- Focus on interventions that decrease health inequity may help address the identified factors that are associated with death in this population. This study provides clinicians with a clearer picture of the potentially injured structures that may lead to death.

## Introduction

Despite commentary and policy focus over the last 20 years, serious youth violence and knife-related deaths remain a significant public health challenge.^1–3^ In 2024 the UK government announced it aimed to halve knife crime within this parliament.^4^

Analysis of police and hospital data in England and Wales by the Youth Endowment Fund found that for those aged 16–24, there has been a 75% increase in knife-enabled crime between 2012/2013 and 2022/2023, despite knife-related homicide and hospital admissions being relatively static over the last decade.^5^ A 2023 survey of 7500 13–17 years old in England and Wales reported 4% had carried a weapon in the last year.^6^

In England, all children have their deaths reviewed by a statutory, multiagency Child Death Overview Panel (CDOP). On 1 April 2019, the National Child Mortality Database (NCMD) was formed to collate data from CDOPs. It is the first database of its kind to systematically collate all deaths in children from birth to 17 years old.

This analysis aimed to use the data regarding deaths of children and young people (CYP) due to a knife injury, available in the NCMD database, to identify potential strategies to reduce the number of CYP who die from knife injuries. We report the demographics of CYP who have died and report the preinjury, injury and postinjury factors associated with death.

## Methods

### Study design and population

This was a retrospective observational cohort study of all CYP included on the NCMD between 1 April 2019 and 31 March 2024. A detailed description of the NMCD process is reported elsewhere.^7^ All child deaths reported to NCMD are coded by three independent paediatricians to identify the likely category of the cause of death. Any death that had been identified by one or more coders as knife-related was included in this cohort. Cases were extracted on 29 July 2024.

For every death, the NCMD collects demographic data, a suspected cause of death, a narrative account of the circumstances of death and a child death analysis form. Postmortem records, hospital and prehospital medical records and social care records can be collected by the NCMD but are not collected for every death. For this paper, reviewers had access to all data available on the NCMD database for each included case.

### Data extraction

As most cases go through lengthy criminal proceedings, years can elapse between death notification and the finalisation of the CDOP review. Between 2019 and 2024, there were two available cohorts for analysis: a full cohort with available demographic data, and a detailed analysis cohort with full NCMD data available.

The full cohort was available from notification forms for all cases occurring during the studied period, April 2019 to March 2024. Extracted demographic data included age at death, sex at birth, ethnicity and region (defined by the Office of National Statistics (ONS)) in which the death was reported and Index of Multiple Deprivation (IMD) by home address.

A second, detailed analysis cohort consisted of cases where the full NCMD data was available after completion of legal proceedings and the CDOP review. CYP who died up until 28 February 2022, were included. The NCMD was last reviewed for available cases in November 2024. Cause of death determination was based on the cause of death reported to the NCMD following the completion of the child death review process.

Data extraction was an iterative process. One author (TR) reviewed the NCMD forms available from six cases to identify data availability. A draft data collection tool was generated in discussion with other authors (TR, JC, SW, RM, EC) (online supplemental file 1).

Following this, two authors (TR, JC) reviewed a further 25 cases using the draft data collection tool. Based on this review of cases, authors (TR, JC, SW, RM, EC) as a group agreed to stratify data collection into three sections based on the study aims and the available data: preinjury, injury and postinjury factors leading to death. Final agreed data collection items are reported in online supplemental file 2.

### Data analysis

Demographic data for the full cohort are presented descriptively. For key demographic variables, year of death (defined as April to March, starting in April 2019), ethnicity (‘Asian or Asian British’, ‘Black or Black British’, ‘Mixed’, ‘Other’, ‘White’, ‘Unknown/missing’), region of England and deprivation (quintiles of the IMD) rates of death were derived per 100 000 CYP assuming a Poisson distribution. ONS Census data from 2021 was used to derive the population at risk. To assess if the rate of death varied by predefined covariates (year, ethnicity, region, IMD), two Poisson models were compared; one with and one without the covariate, and the likelihood ratio test was used to compare for a difference in fit. For ordinal variables (Year and IMD), an OR for each increase in the variable was also derived. For all variables, an incidence rate ratio (IRR) was reported. All analysis was performed using Stata V.18 (StataCorp).

Preinjury data were coded using three distinct frameworks. Cases were coded for items of the adverse childhood experiences (ACEs) framework which were specifically focused on adversity within the child’s home context.^8^ Categories of emotional and physical neglect were coded under one item as the case files commonly mentioned ‘neglect’ without specific details. Case files were then coded using Hamby *et al’s* model of polyvictimisation, focusing on experiences of adversity outside of the home context, relevant to the wider context of public space ‘knife crime’^9^ and finally examined to report onward support referral pathways for CYP. Rates were reported descriptively.

Injury data is classified by body region using methodology described previously.^8^ Data from postmortem reports were extracted to report the number of fatal injuries sustained and the number of anatomical structures fatally injured by each fatal wound. Patterns of injuries causing death are stratified by age (less than 10 years of age and 10 years of age and older). Data is presented using upset plots. The total number and location of all injuries sustained was extracted from postmortems. The median number of fatal and non-fatal injuries is reported descriptively and by covariates (year, ethnicity, region, IMD). Where postmortem reports were missing, this is reported.

Postinjury data is reported descriptively. Data items analysed focused on the location of death and the interventions delivered. During data extraction, it was noted that there was significant regional and case variance in the clinical information provided. Therefore, it was decided (by SW, TR, JC) that intervention data was to be taken from postmortem reports only, as these demonstrated more uniformity. Blood product administration was taken from available clinical data.

### Patient and public involvement

We did not directly include patient and public involvement in this study, but the NCMD has many key patient groups which support its work and aims, including support database development, project ideas and dissemination.

## Results

Between April 2019 and March 2024, there were 145 cases of CYP who died due to knife injuries. Of these, 40% (n=58) had a completed CDOP review and full dataset uploaded to the NCMD (online supplemental file 6).

### Demographics (n=145)

**Figure 1:**
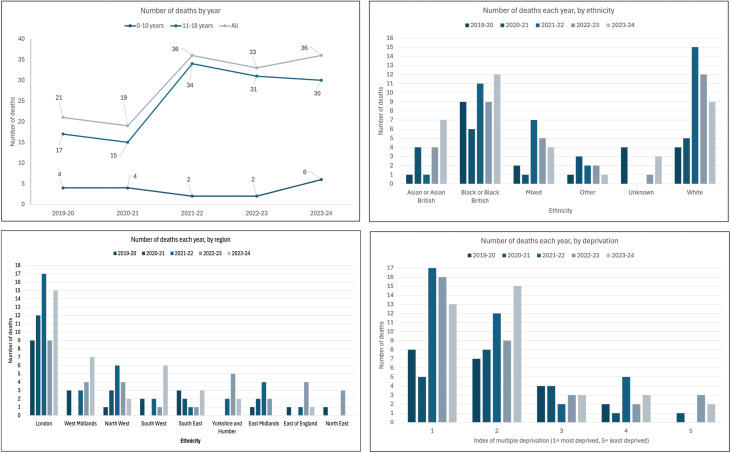
Trends of death between 2019 and 2024.

The mean age of death was 14.4 years (n=145, SD 4.2) and sex was reported as male in 90.3% (n=131) of cases (table 1). Of the deaths, CYP were of ‘Black or Black British’ and ‘White’ ethnicities in 32.4% (n=47) and 31.0% (n=45) of cases, respectively. Figure 1 shows the number of annual deaths, stratified by age at death (0–10 years, and 11–17 years), ethnicity, region and IMD. Three quarters of deaths (75.9%, n=110) were from the lower two quintiles of deprivation (figure 1). London was the region where 42.8% (n=62) of all deaths occurred.

**Table 1:** Demographics of the full and detailed analysis cohorts

	Full cohort		Detailed analysis cohort	
**Age**	**n=145**		**n=58**	
Age at death, median, IQR Age at death, mean, SD	16 (15–17) 14.4 (4.2)		16 (15–17) 14.1 (4.5)	
	**%**	**n**	**%**	**n**
Age<5 years	6.2	9	8.6	5
5–10 years	6.2	9	5.2	3
11–14 years	8.3	12	10.3	6
15–17 years	79.3	115	75.9	44
Missing	0	0	0	0
Sex	**%**	**n**	**%**	**n**
Male	90.3	131	93.1	54
Female	9.0	13	6.9	4
Missing	0.7	1	0	0
Ethnicity	**%**	**n**	**%**	**n**
Asian or Asian British	11.7	17	10.3	6
Black or Black British	32.4	47	36.2	21
Mixed	13.1	19	10.3	6
Other	6.2	9	6.8	3
White	31.0	45	31.0	18
Unknown/missing	5.5	8	5.2	3
Region of CDOP			**%**	**n**
East Midlands	6.2	9	12.1	7
East of England	4.8	7	3.4	2
London	42.8	62	48.3	28
Northeast	2.8	4	1.7	1
Northwest	11.0	16	10.3	6
Southeast	6.9	10	10.3	6
Southwest	7.8	11	1.7	1
West Midlands	11.7	17	8.6	5
Yorkshire and the Humber	6.2	9	3.4	2
Socio-economic status, IMD quintile by LSOA of home address	**%**	**n**	**%**	**n**
1 (most deprived)	40.7	59	39.7	23
2	35.2	51	37.9	22
3	11.0	16	13.8	8
4	9.0	13	6.9	4
5 (least deprived)	4.1	6	1.7	1

CDOP, Child Death Overview Panel; IMD, Index of Multiple Deprivation; LSOA, lower layer super output area.

**Figure 2:**
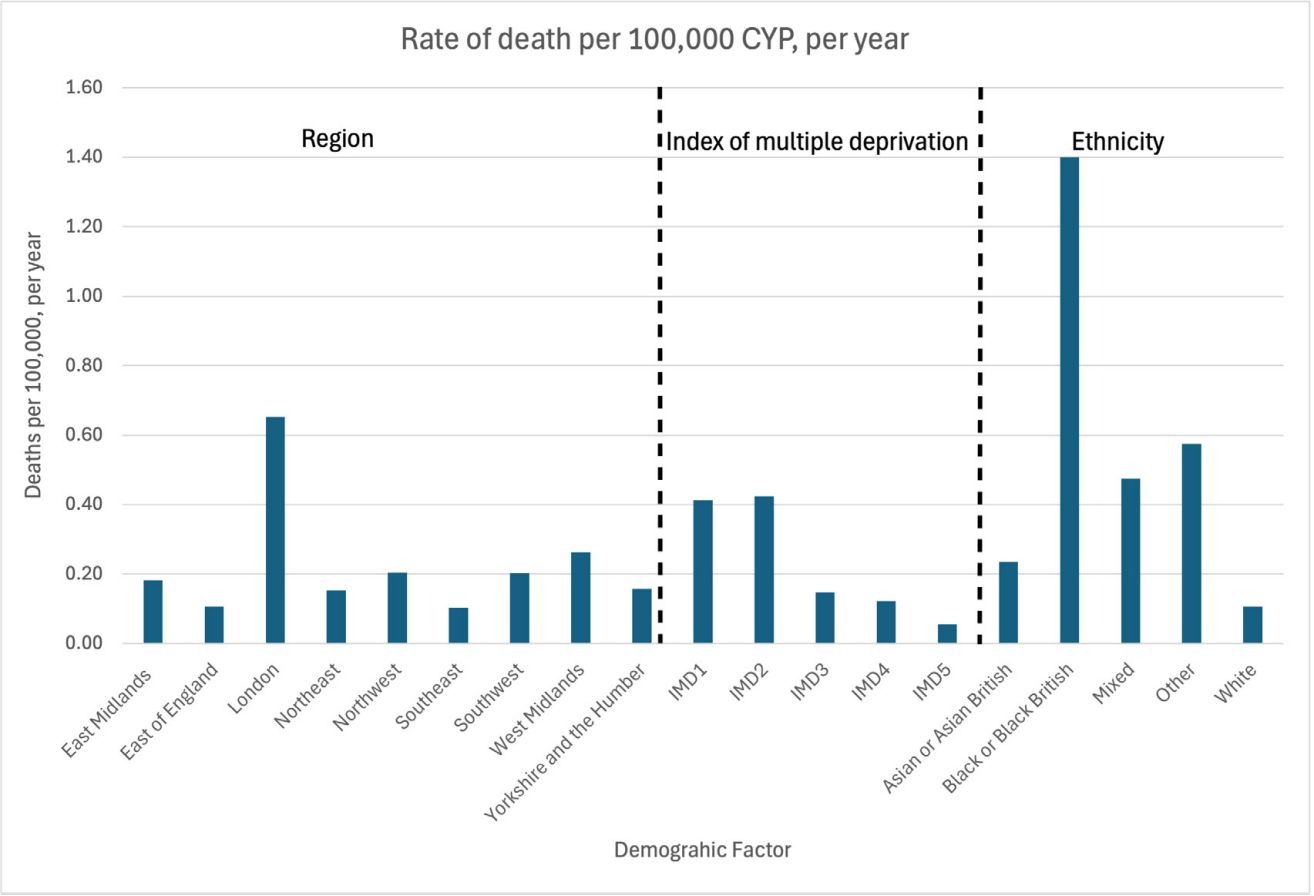
Deaths per 100 000 CYP per year, by region, deprivation and ethnicity. CYP, children and young people; IMD, Index of Multiple Deprivation.

Rate of death by knife injuries varied by year (p=0.0398) ethnicity (p<0.001), region (p<0.001) and IMD (p<0.001) (table 2, figure 2). Rates of death increased with time (IRR per year 1.17 (95% CI 1.04 to 1.31), p=0.010), with the highest rate seen in 2023–2024.

**Table 2:** The IRR by ethnicity, region, IMD and region

Variable	N	Rate per 100 000 CYP deaths, per year (95% CI)	IRR (95% CI)	p_difference†_	p_trend*_
Ethnicity					
Asian or Asian British	17	0.23 (0.14 to 0.38)	2.23 (1.27 to 3.89)	<0.001	N/A
Black or Black British	47	1.40 (1.03 to 1.86)	13.29 (8.23 to 20.00)		
Mixed	19	0.47 (0.29 to 0.74)	4.50 (2.63 to 7.69)		
Other	9	0.58 (0.26 to 1.09)	5.46 (2.67 to 11.17)		
White	45	0.11 (0.08 to 0.14)	1.00 (Ref)		
Missing	8				
Region					
East Midlands	9	0.18 (0.08 to 0.34)	0.28 (0.14 to 0.56)	<0.001	N/A
East of England	7	0.11 (0.04 to 0.22)	0.16 (0.07 to 0.35)		
London	62	0.65 (0.50 to 0.84)	1.00 (Ref)		
Northeast	4	0.15 (0.04 to 0.39)	0.23 (0.08 to 0.64)		
Northwest	16	0.20 (0.12 to 0.33)	0.31 (0.18 to 0.54)		
Southeast	10	0.10 (0.05 to 0.19)	0.16 (0.08 to 0.31)		
Southwest	11	0.20 (0.10 to 0.36)	0.31 (0.16 to 0.59)		
West Midlands	17	0.26 (0.15 to 0.42)	0.40 (0.24 to 0.69)		
Yorkshire and the Humber	9	0.16 (0.07 to 0.30)	0.24 (0.12 to 0.48)		
Missing	0				
IMD quintile by LSOA of home address					
1 (most deprived)	59	0.41 (0.31 to 0.53)	7.46 (3.22 to 17.29)	<0.001	<0.001
2	51	0.42 (0.32 to 0.56)	7.68 (3.29 to 17.89)		
3	16	0.15 (0.08 to 0.24)	2.65 (1.04 to 6.78)		
4	13	0.12 (0.06 to 0.21)	2.20 (0.84 to 5.79)		
5 (least deprived)	6	0.06 (0.02 to 0.12)	1.00 (Ref)		
Missing	0				
Year of death					
2019–2020	21	0.18 (0.11 to 0.27)	1.00 (Ref)	0.0398	0.010
2020–2021	19	0.16 (0.10 to 0.25)	0.90 (0.49 to 1.68)		
2021–2022	36	0.31 (0.21 to 0.42)	1.71 (1.00 to 2.94)		
2022–2023	33	0.28 (0.19 to 0.39)	1.57 (0.91 to 2.72)		
2023–2024	36	0.31 (0.21 to 0.42)	1.71 (1.00 to 2.94)		
Missing	0				

* ‘ptrend’ is the evidence for a trend in death rates across the ordinal variables (eg, year or deprivation).

† ‘pdifference’ is the evidence that the rates of death are different across the characteristic (eg, across age groups).

CYP, children and young people; IMD, Index of Multiple Deprivation; IRR, incidence rate ratio; LSOA, lower layer super output area; N/A, not assessed.

Children of ‘Black or Black British’ ethnicity had the highest rate of death at 1.40 (95% CI 1.03 to 1.86) per 100 000 CYP, per year, while the lowest rate was seen in children of ‘White’ ethnicities (0.11 (95% CI 0.08 to 0.14)) (table 2). Compared with CYP of ‘White’ ethnicity, the IRR for ‘Black or Black British’ CYP was 13.29 (95% CI 8.23 to 20.00), indicating a 13 times higher risk of death.

London had the highest rate of deaths per 100 000 CYP, per year (0.65; 95% CI 0.50 to 0.84), followed by the West Midlands (0.26; 95% CI 0.15 to 0.42). The lowest rates were seen in the Southeast (0.10 (95% CI 0.04 to 0.39)) and the East of England (0.11 (95% CI 0.04 to 0.22). Compared with London, the IRR for the West Midlands was 0.40 (95% CI 0.24 to 0.69), indicating half the risk of death outside of London.

CYP living in the most deprived areas of England had the highest rates of death per 100 000 CYP per year, 0.41 (95% CI 0.31 to 0.53) and 0.42 (95% CI 0.32 to 0.58), respectively. compared with CYP living in the least deprived areas, the IRR for death was 7.48 (95% CI 3.22 to 17.29) for children living in the most deprived areas, indicating a greater than seven times higher risk of death for CYP.

Repeating the analysis stratified by region (London vs England (without London)), and by ethnicity, suggested that rates of death per 100 000 CYP, per year, were highest for CYP of ‘Black or Black British’ ethnicity living in London (1.81 (95% CI 1.21 to 2.60)). The rate was 0.25 (95% CI 0.12 to 0.46) per 100 000 CYP deaths, per year for ‘White’ ethnicity in London. The lowest rates were seen in children of ‘White’ ethnicities living outside of London (0.09 (95% CI 0.07 to 0.13)) (online supplemental file 3).

### Detailed analysis of deaths

The analysis of preinjury, injury and postinjury factors relates to the cohort of 58 CYP for which a full NCMD file was available for analysis. Demographics are presented in table 1.

One death was excluded from the preinjury analysis, except for the perpetrator analysis, as the death related to a neonate who died after a perimortem caesarean section performed on the mother secondary to a stab wound to the mother’s chest.

### Preinjury factors (n=57)

#### Preinjury adverse childhood experiences (n=57)

The most common ACE was a documented history of domestic violence and abuse (DVA) which was present in 57.9% (n=33) of cases. Children experienced physical abuse at home in 37.0% (n=21) of cases and 1.7% (n=1) of CYP had experienced documented sexual abuse. The loss of a key adult figure (through separation or bereavement) was documented in 50.9% (n=29) of cases. A quarter of children (24.5%, n=14) resided with an adult with mental illness, and nearly a third (31.57%, n=18) lived in a household with substance abuse. Neglect was experienced by 17.5% (n=10) of CYP and 8.8% (n=5) experienced emotional abuse. (table 3).

**Table 3:** Prevalence of preinjury risk factors presence in case files of CYP who died of knife injuries

	%	N=57
Adverse childhood experiences		
Neglect (emotional and/or physical)	17.5	10
Loss of key figure (separation, divorce, bereavement)	50.9	29
Mental illness in household	24.6	14
Household substance abuse	31.6	18
Domestic violence and abuse (‘mother treated violently’)	57.9	33
Criminal household member (prison)	5.3	3
Emotional abuse	8.8	5
Physical abuse	36.8	21
Sexual abuse	1.8	1
Polyvictimisation		
Knife carrying in case files	24.6	14
‘Gang-involved’ in case files	36.8	21
Involvement in the use and/or supply of illicit substances	68.4	39
Concerns raised about child criminal exploitation in the case files	52.6	30
Children recorded as both victims and perpetrators of violence in childhood	59.6	34
Preinjury intervention pathways for children		
Social care		
Never/not known to social care	24.6	14
Previously known but not an open case	42.1	24
Unknown	1.8	1
Known to social care at the time of death:	33.3	19
On a child protection plan	–	*6*
Look after child	–	*4*
Child in need	–	*11*
Other	–	*3*
School (school aged CYP, n=53)		
Child experienced periods of school exclusion	43.9	25
DVA specialist support		
Referral to adult DVA services	7.0	4
Referral to child specific DVA support service	0.0	0
Mental health and/or neurodiversity		
Neurodiversity and/or mental health concern mentioned in NCMD form	50.9	29
Child referred to CAMHS	28.1	16
Child on an EHCP	5.3	3
Attention deficit hyperactivity disorder suspected and/or diagnosed	15.8	9
Speech and language therapy referral	5.3	3

CAMHS, Child and Adolescent Mental Health Services; CYP, children and young people; DVA, domestic violence and abuse; EHCP, educational health and care plans; NCMD, National Child Mortality Database.

#### Preinjury polyvictimisation

Concerns around child criminal exploitation were mentioned in 51.7% (n=30) of the child-death review case notes reviewed and almost half of children were viewed as being victimised within a broader context of serious youth violence (45.6%, n=26). Gang involvement is difficult to substantiate, but it was mentioned in 36.8% (n=21) of case files. The use, or dealing, of illegal substances was reported in 68.4% (n=39) of CYP prior to death. Concerns around the child carrying knives were recorded in a quarter (25.5%, n=14) of case files. Over half of the cohort (59.6%, n=34) were recorded as being both victims and perpetrators of violence in childhood.

#### Preinjury intervention pathways

Of the 57 cases, 33.3% (n=19) had active involvement from social services at the time of death and a further 42.1% (n=24) had been previously known to these services. Despite the high prevalence of DVA, none (0% (n=0)) received child-specific DVA support; however, 7.0% (n=4) were offered DVA interventions adjoined to the non-abusing parent. Support referrals for concerns related to neurodiversity or mental health prior to death were mentioned in 50.9% (n=29) of cases and 27.6% (n=16) of children had been referred to Child and Adolescent Mental Health Services. There were 53 school-aged children (aged 5 years or older), of whom almost half (n=25, 47.2%) had been excluded from school for periods prior to their death.

#### Preinjury: perpetrator (n=58)

All 58 cases were included in the perpetrator analysis, including the neonatal death. Within the NCMD data, limited data about the perpetrator is available. Of the 58 cases, the perpetrator was known to the victim but unrelated in 39.7% (n=23), a biological parent in 10.3% (n=6), was a stranger in 3.4% (n=2) and the relationship was unknown or undocumented in 46.6% (n=27) of cases.

### Injury factors (n=55)

#### Injuries

Of the 58 cases available for analysis, two postmortems were unavailable and one (a neonate) was excluded as the knife injuries pertained to the mother.

Of the 55 cases in which postmortem data was available, 69.1% (n=38) were reported to have died of a single fatal wound, with a median of 3.0 (IQR 1.3–6.00) total knife wounds. Two fatal injuries were reported in 20.0% (n=11) of CYP, with a median of 6.0 (IQR 3.0–13.5) knife injuries, 5.5% (n=3) died of three fatal wounds and 5.5% (n=3) died of more than three fatal wounds. In total, 87.2% (n=48) of victims had multiple stab wounds, with a median number of injuries of 4.0 (IQR 2.0–8.5). The median number of fatal and overall injuries reported by region, ethnicity and IMD is reported in the online supplemental file 4.

The cause of death was reported as stab wounds to the chest in 67.3% (n=37) of cases, (this included four cases who died of hypoxic brain injury secondary to chest wounds). Stab wounds to the neck were the cause in 12.1% (n=7) of deaths. This is further detailed in online supplemental file 7.

**Figure 3:**
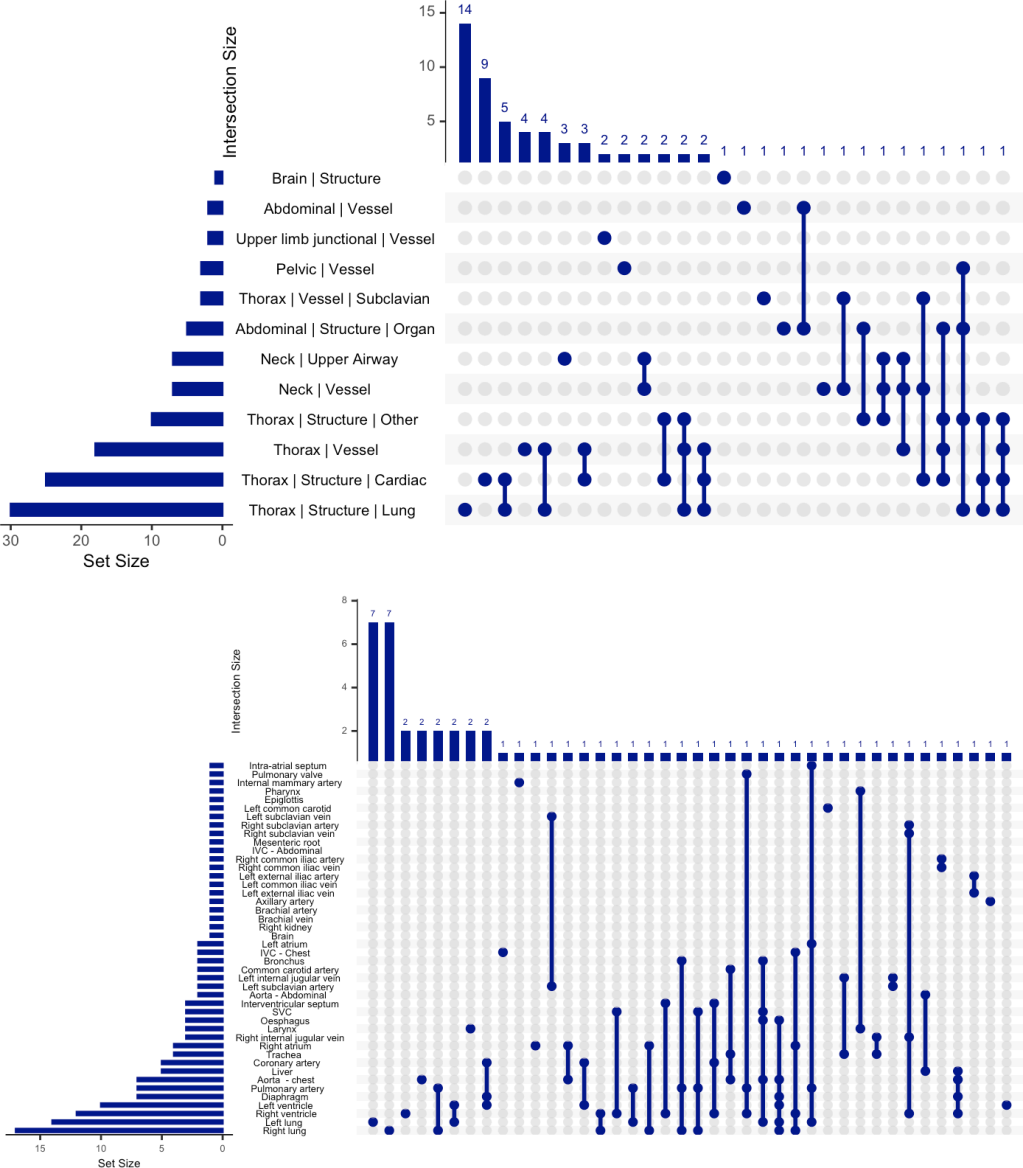
(a and b) Anatomical structures reported as fatally injured in postmortems. Each dot represents an anatomical structure reported as fatally injured in a postmortem report. Each set of dots, joined by a line, represents a combination of structures damaged by one wound. The bar charts represent the frequency of those individual or combination of injuries. IVC, inferior vena cava, SVC, superior vena cava.

A detailed description of the different anatomical structures injured and reported to have led to death is presented in figure 3a and b. Figure 3a groups structures by anatomical location and demonstrates that thorax and neck structures are the most frequently damaged structures. Figure 3b outlines the number of different anatomical structures reported to have led to death. No deaths were attributed to external compressible haemorrhage.

Injury patterns for the cases where CYP were under 10 years of age are not reported separately for confidentiality.

#### Postinjury (n=58)

Of the 58 CYP included in the detailed analysis, 60.3% (n=35) died in the community, 24.1% (n=14) died in an emergency department, 10.3% (n=6) died in a critical care setting, 3.4% (n=2) died in an operating theatre and 1.7% (n=1) died on a hospital ward.

In terms of interventions, 37.9% (n=22) received a blood transfusion and 41.3% (n=24) received advanced vascular access (defined as subclavian, internal jugular or femoral vascular access). An advanced airway was placed in 72.4% (n=42) of cases (32 endotracheal tubes, 9 supraglottic airways and 1 surgical airway). A thoracotomy was performed in 56.9% (n=33) of cases. The location of death and rates of thoracotomy reported by region, ethnicity and IMD are reported in the online supplemental file 5.

## Discussion

### Key findings

While knife-related mortality in the 16–24 years old age group has remained relatively static over the past decade,^5^ this study finds that deaths among those aged 0–17 years have increased over the period studied (2019–2023). The findings indicate that the group at highest risk of experiencing a fatal stab wound in England are CYP of ‘Black/Black British’ ethnicity who reside in urban areas of deprivation. Many of the CYP that died were exposed to ACEs before death and were well known to statutory services. A history of DVA was identified in more than half the children that died. In terms of injuries sustained, the majority of CYP (60.3% (n=35)) died in the prehospital setting after receiving injuries to anatomical structures in the thorax.

### In context

This intersecting position of race, class, gender and poverty can situate young people in a position of marginalisation. Marginalisation of this cohort is well established, where research on adultification bias has exposed the ways in which poor black children are more likely to be seen as less vulnerable and therefore, ‘at a heightened risk of their safeguarding needs being unmet’.^9^

The CYP in this dataset were likely to experience a wide range of ACEs. Most were involved with statutory services prior to injury, constituting possible intervenable points prior to death. As CYP reached adolescence, many fit a model of polyvictimisation of violence and marginalisation.^10 11^ Most CYP were exposed to DVA prior to their death, without recourse to specific support. Experiencing unsupported adversity increased susceptibility to criminal exploitation and violence. These findings broadly concur with global trends in preinjury risk factors as reported by the WHO.^12^ Policy interventions that decrease ACEs and promote recovery have the potential to prevent future deaths.

The patterns of injury sustained in CYP, and the location of death, suggest that in the majority of cases rapid blood loss from injuries to structures predominantly in the chest leads to death and that management in the prehospital setting is required in most cases to prevent death. While every child who died is entered onto NCMD and had a postmortem performed, detailed analysis of prehospital interventions undertaken and timings was not possible due to regional variations in data submitted to NCMD. Improving prehospital data collection in the NCMD database and aligning data collected with other available databases presents an opportunity for further development.

In the cases where data was available, it is clear, CYP received advanced medical interventions aimed at reversing causes of traumatic cardiac arrest, including advanced airway and surgical procedures. Evidence to support, or refute, critical interventions in traumatic cardiac arrest and the timings of these in CYP and adolescents is lacking.^13^ Improved understanding of outcomes following traumatic cardiac arrest, stratified by age, is required, as there are suggestions outcomes are worse for CYP 15 years and under, compared with those over 15 years old.^14^

A deeper understanding of the lethality of structures damaged, and when and where these injuries become fatal, will enable advancement in management and clinical innovation following penetrating trauma to improve survival. This study provides emergency clinicians both in and out of hospital, as well as damage control surgeons, with a clearer picture of the potential anatomical structures that may lead to death. Improved data collection from point of injury through to in-hospital care will allow for a better understanding of the impact of postinjury interventions on outcomes.

### Limitations

This study is limited to children fatally injured only and does not include those with severe injuries which were not fatal. Therefore, the results are not generalisable to those with non-fatal injuries. The results are limited by the limitations in data collected as part of the child death review process. This is particularly notable in terms of limiting detailed analysis of prehospital interventions.

This study highlights the importance of improving preinjury identification and support for children who are experiencing marginalisation and adversity. Our findings highlight the need for services to examine childhood experiences of violence and abuse both inside and outside their homes, and to confront the ways in which certain children from racialised and poor backgrounds may be overlooked for early intervention. From our sample, despite frequent contact with statutory services, many children lacked targeted support for ACEs, particularly DVA. This revealed critical gaps in early intervention.

## Supplementary material

10.1136/emermed-2025-215154 online supplemental file 1

10.1136/emermed-2025-215154 online supplemental file 2

10.1136/emermed-2025-215154 online supplemental file 3

10.1136/emermed-2025-215154 online supplemental file 4

10.1136/emermed-2025-215154 online supplemental file 5

10.1136/emermed-2025-215154 online supplemental file 6

10.1136/emermed-2025-215154 online supplemental file 7

## Data Availability

Data are available upon reasonable request.
