# Supplementary material for: Preinjury, injury and post-injury factors leading to death in children and young people who were victims of knife crime in England between 2019 and 2024: a review of the National Child Mortality Database
Source: Emerg Med J. 2026 Apr 20;43(8):e215154. doi: 10.1136/emermed-2025-215154 (PMC13479297; doi:10.1136/emermed-2025-215154)
Supplement: Supplementary file 1 [file emermed-43-8-s001.pdf]

NCMD FORM
Sex
Date of birth
home address postcode
date of death
time of death
where was the child when they died
suspected cause of death
mode of death
notification details
what was the cause of death as given on the MCCD, or the coroner's conclusion as to the cause of death, if known
what subcategory does this child's death fall into?
were any of the following historic risk factors present in this case?
were any of the following risk factors identified in relation to the events surround the death?
were any of the following risk factors identified in relation to the child's parent/carer
was there any evidence of any intent to kill or harm the child
please provide details
was there any apparent attempt to conceal or hide the circumstance's of the child's death?
was there any evidence of neglect pre-dating the fatal incident
please give details
was there any evidence of physical abuse/inflicted injury pre dating the fatal incident
please give details
what was the method of homicide
how may alleged perpetrators were involved in this incident?
relationship to alleged perpetrator(s) to child
age of perpetrator(s)
were the alleged perpetrators using drugs and/or alcohol at the time of the incident
were the alleged perpetrators known to police for durg or alcohol related offences?
were the alleged perpetrators known to the polcie for any other offences?
were the alleged perpetrators known to be violent
were the alleged perpetrators know to mental health services
were there any other possible victims
did the alleged perpetrator take/attempt to take their own life?

was the incident related to gang violence?
was there a criminal trial
if yes what was the nature of the conviction
how long after initial presentation to medical attention was the child declared dead?
was there any indication that the child/child's family lived in poverty and/or deprivation
was there known domestic violence/abuse in the household?
was the child known to children's social care prior to their death/the event leading to their death?
please provide details
factors in the social environment including parenting capacity
factors in the physical environment
was the child known to mental health services (child and adolescent or adult mental health services)
were there any issues relating to the treatment or healthcare management plan
please provide details
were there organisational issues that may have contributed to the child's vulnerability, ill-health or death?
please provide details

Pre-injury items

Intersectional marginalization	
Childhood DVA & other male perpetrated violence	
DVA specific support offered/taken?	
School based violence/ aggression	
On road	Exploitation
	Drugs
	Periods of school exclusion
	On-road/Gangs/ SOC
	Neurodiversity/ Mental health

Injury details

Post-Mortem	date of death
	time of death
	Cause of death 1a
	Cause of death 1b

	Cause of death 1c
	Cause of death II
	History
	number of injuries
	description of significant injuries relating to death
	PM conclusions

Pre-hospital data	Time of injury
	nature of injuries when call first made
	arrival of first healthcare professional
	interventions provided
	arrival of HEMS/Critical Care
	injuries identified in the pre-hospital setting
	death in the pre-hospital setting?
	arrival at hospital
Hospital data	Time of arrival at hospital
	nature of injuries suspected on arrival
	interventions provided
	imagining results
	results of surgery
	time of death
	date of death
	proposed cause of death in hospital
