# Supplementary material for: Preinjury, injury and post-injury factors leading to death in children and young people who were victims of knife crime in England between 2019 and 2024: a review of the National Child Mortality Database
Source: Emerg Med J. 2026 Apr 20;43(8):e215154. doi: 10.1136/emermed-2025-215154 (PMC13479297; doi:10.1136/emermed-2025-215154)
Supplement: Supplementary file 2 [file emermed-43-8-s002.pdf]

Id
WhenCaseReviewedByResearchers
Sex
DateOfBirth
DateOfDeath
TimeOfDeath
AgeAtDeath
Region
IMD_Decile
PlaceofDeath
WhereAtOnsetOfIllnessOrIncident
SuspectedCauseOfDeath
NotificationDetails
ModeofDeath
CauseOfDeathMCD1A1B1C2
SupplementViolent_SubCategory
SupplementViolent_HistoricRiskFactors_MF
SupplementViolent_RiskFactorsRelatingToEvent_MF
SupplementViolent_RiskFactorsRelatingToParentCarer_MF
SupplementViolent_IntentToKillOrHarm
SupplementViolent_IntentToKillOrHarmDetails
SupplementViolent_NeglectPreFatalIncident
SupplementViolent_NeglectPreFatalIncidentDetails
SupplementViolent_EvidenceOfAbusePreFatalIncident
SupplementViolent_EvidenceOfAbusePreFatalIncidentDetails
SupplementViolent_HomicideMethod
SupplementViolent_NumberOfAllegedPerpetrators
SupplementViolent_RelationshipofAllegedPerpetratorToChild_MF
SupplementViolent_AgeOfAllegedPerpetrator
SupplementViolent_PerpUsingDrugsAlcoholAtTimeOfIncident
SupplementViolent_PerpKwnoToPoliceForDrugAlcoholOffences
SupplementViolent_PerpKnownToPoliceOtherOffences
SupplementViolent_PerpKnownToBeViolent
SupplementViolent_PerpKnownToMentalHealthServices
SupplementViolent_OtherPossibleVictims
SupplementViolent_PerpAttemptSuicide
SupplementViolent_IncidentRelatedToGangViolence
SupplementViolent_CriminalTrial
SupplementViolent_NatureOfConviction_MF
SupplementSUDI_HowLongAfterMedicalPresentationDeclaredDead
SocialEnvironmentDeprivation

**HouseholdViolence**	
**KnownToSocialCare_Summary**	
**SC_CPP**	
**SC_LAC**	
**SC_CIN**	
**SC_Other**	
**KnownToCAMHS**	
**TreatmentIssues**	
**TreatmentIssuesDetails**	
**OrganisationIssues**	
**OrganisationIssuesDetails**	
**Child_ContributoryFactors**	
**SocialEnvironment_ContributoryFactors**	
**PhysicalEnvironment_ContributoryFactors**	
**ServiceProvision_ContributoryFactors**	
**PrimaryCategoryofDeath_CDOP**	
**CDOPCauseOfDeath**	
**Column1**	
**EthnicGroup**	
**Data extract**	
Case number	
Intersectional marginalization	
Childhood DVA & other male perpetrated violence	
DVA specific support offered/taken?	
School based violence/ aggression	
On road	Exploitation
	Drugs
	Periods of school exclusion
	On-road/Gangs/ SOC
	Neurodiversity / Mental health
date of death	
time of death	
PM Available	
Cause of death 1a	
Cause of death 1b	
Cause of death 1c	

Cause of death II
number of injuries post mortem report states could have contributed to death
Site of death injury 1 (Head/neck - Chest - UL junctional (axilla or shoulder) - Upper limb - Abdomen - LL junctional (groin, buttock, perineum) - Lower limb
more specific death injury 1 (e.g subclavian artery dissection, tracheal laceration)
Head: Brain Parenchyma, IV bleeding and SAH
Limb: Axillary A
Limb: Ext Iliac A and V
Abd: Liver
Abd: Liver Blood Vessels
Common iliac Artery and Vein (Right)
Abd: Diaphragm
Abd: IVC
Abd: Aorta
Heart:LA
Heart:RA
Heart: LV
Heart: RV
Heart: Coronary Artery
Heart: RV outflow tract
Heart: IV septum
Heart Aortic Valve
Heart Mitral Valve
Heart Pericardium
Chest IVC
Chest SVC
Chest Aorta
Chest Oesophagus
Chest Trachea
Chest: Int Mammary A
Lung Parenchyma
Lung: Pulmonary Artery
Lung: Right Main Bronchus
Neck Trachea
Neck Oesophagus
Neck: Internal Jugular Vein

Neck: Pharynx
Neck :Carotid Artery
Neck:Larynx
Neck: Subclavian A
Neck Subclavian V
Site of death injury 2 (Head/neck - Chest - UL junctional (axilla or shoulder) - Upper limb - Abdomen - LL junctional (groin, buttock, perineum) - Lower limb
more specific death injury 2 (e.g subclavian artery dissection, tracheal laceration)
Heart RV
Heart Pericardium
Chest SVC
Lung: Parenchyma
Lung: Intercostal Vessels
Lung: Pulmonary Artery
Lung: 2nd to 3rd order bronchioles
Neck:Larynx
Abd: Diaphragm
Abd Colon
Abd Retroperitoneal Space
Abd: Liver
Site of death injury 3 (Head/neck - Chest - UL junctional (axilla or shoulder) - Upper limb - Abdomen - LL junctional (groin, buttock, perineum) - Lower limb
more specific death injury 3 (e.g subclavian artery dissection, tracheal laceration)
Brachial A and V
Chest Aorta
Chest: Diaphragm
Chest: Lung Parenchyma
Abd: Liver
Abd: Kidney
Abd: Retroperitoneal Space
Abd: Common Iliac Vein
Abd: Colon
Structure 1 that caused death
PM conclusinos
all wounds (regions)
Head/Face

Neck
Chest Ant
Chest Posterior
Abd Anterior
Abd Posterior
UL Junctional
UL
LL Junctional
LL
Notes
Interventions
**Case number**
**Anatomical location of injury**
**Prehospital data there**
**Died at scene**
**Thoracotomy**
**Location of thoractomy**
**Thoracostomy R**
**Thoracostomy L**
**Blood**
**Units**
**IO**
**Subclavian access**
**Central line**
**Chest seal**
**Needle decompression**
**Hilar twist**
**Laparotomy**
**location of laparotomy**
**Theatre**
**ECMO**
**Tourniquet**
**Resuscitative hysterotomy**
**Comments**
