# Supplementary material for: Preinjury, injury and post-injury factors leading to death in children and young people who were victims of knife crime in England between 2019 and 2024: a review of the National Child Mortality Database
Source: Emerg Med J. 2026 Apr 20;43(8):e215154. doi: 10.1136/emermed-2025-215154 (PMC13479297; doi:10.1136/emermed-2025-215154)
Supplement: Supplementary file 3 [file emermed-43-8-s003.pdf]

Online supplement – Rates of CYP deaths, per 100,000, per year, in London and England, excluding London		
**Variable**	**N**	**Rate per 100,000 CYP deaths, per year (95% CI)**
**Ethnicity In London**		
Asian or Asian British	7	0.32 (0.13-0.66)
Black or Black British	29	1.81 (1.21-2.60)
Mixed	6	0.54 (0.20-1.17)
Other	8	1.25 (0.54-2.46)
White	10	0.25 (0.12-0.46)
Missing/unknown	2	
**Ethnicity in England, excluding London**		
Asian or Asian British	10	0.20 (0.09 -0.36)
Black or Black British	18	1.02 (0.61-1.52)
Mixed	13	0.45 (0.24 – 0.77)
Other	1	0.11 (0.00 – 0.60)
White	35	0.09 (0.06 – 0.13)
Missing/unknown	6	
