# Supplementary material for: Preinjury, injury and post-injury factors leading to death in children and young people who were victims of knife crime in England between 2019 and 2024: a review of the National Child Mortality Database
Source: Emerg Med J. 2026 Apr 20;43(8):e215154. doi: 10.1136/emermed-2025-215154 (PMC13479297; doi:10.1136/emermed-2025-215154)
Supplement: Supplementary file 4 [file emermed-43-8-s004.pdf]

		Number of fatal injuries	Number of overall injuries
**Ethnicity (n=58)**	**n**	Median (IQR)	Median (IQR)
Asian or Asian British	**6**	1.5 (1-2.75)	3 (1-9.5)
Black or Black British	**20**	1 (1-1)	2 (2-7)
Mixed	**6**	1.5 (1-2)	6.5 (4.5-10.75)
Other	**4**	NR	NR
Unknown	**3**	NR	NR
White	**16**	1 (1-2)	4.5 (2-7)
Excluded/PM not available	**3**	-	-
**Region (n=58)**			
East Midlands	**7**	1 (1-2.5)	13 (4-18.5)
East of England	**2**	NR	NR
London	**27**	1 (1-1)	3 (2-7)
North East	**1**	NR	NR
North West	**5**	1 (1-3)	7 (6-7)
South East	**5**	1 (1-1)	2 (1-7)
South West	**1**	NR	NR
West Midlands	**5**	2 (1-2)	13 (4-18)
Yorkshire and Humber	**2**	NR	NR
Excluded/PM not available	**3**	-	-
**IMD (n=58)**			
1	**23**	1 (1-2)	6 (2.5-11.5)
2	**20**	1 (1-2)	4 (2-10.75)
3	**8**	1 (1-1)	2 (1-4.75)
4	**3**	NR	NR
5	**1**	NR	NR
Excluded/PM not available	**3**		
