# Supplementary material for: Preinjury, injury and post-injury factors leading to death in children and young people who were victims of knife crime in England between 2019 and 2024: a review of the National Child Mortality Database
Source: Emerg Med J. 2026 Apr 20;43(8):e215154. doi: 10.1136/emermed-2025-215154 (PMC13479297; doi:10.1136/emermed-2025-215154)
Supplement: Supplementary file 5 [file emermed-43-8-s005.pdf]

		Location of death						Thoracotomy performed	
		Community n= 31		Hospital n=26		Missing n=1			
**Ethnicity (n=58)**	**n**	n	%	n	%	n	%	n	%
Asian or Asian British	**6**	2	33.3%	4	66.7%	0	0.0%	3	50.0%
Black or Black British	**21**	18	85.7%	3	14.3%	0	0.0%	16	76.2%
Mixed	**6**	2	33.3%	4	66.7%	0	0.0%	3	50.0%
Other	**4**	2	50.0%	2	50.0%	0	0.0%	3	75.0%
Unknown	**3**	1	33.3%	2	66.7%	0	0.0%	2	66.7%
White	**18**	6	33.3%	11	61.1%	1	5.60%	6	33.3%
**Region (n=58)**									
East Midlands	**7**	3	42.9%	4	57.1%	0	0.0%	3	42.9%
East of England	**2**	1	50.0%	1	50.0%	0	0.0%	1	50.0%
London	**28**	9	32.1%	19	67.9%	0	0.0%	18	64.3%
North East	**1**	1	100.0%	0	0.0%	0	0.0%	1	100.0%
North West	**6**	5	83.3%	1	16.7%	0	0.0%	2	33.3%
South East	**6**	2	33.3%	3	50.0%	1	16.7%	1	16.7%
South West	**1**	1	100.0%	0	0.0%	0	0.0%	1	100.0%
West Midlands	**5**	2	40.0%	3	60.0%	0	0.0%	5	100.0%
Yorkshire and Humber	**2**	2	100.0%	0	0.0%	0	0.0%	1	50.0%
**IMD (n=58)**									
1	**23**	10	43.5%	13	56.5%	0	0.0%	16	69.6%
2	**22**	13	59.1%	9	40.9%	0	0.0%	12	54.5%
3	**8**	6	75.0%	2	25.0%	0	0.0%	2	25.0%
4	**4**	1	25.0%	2	50.0%	1	25.0%	3	75.0%
5	**1**	1	100.0%	0	0.0%	0	0.0%	0	0.0%
