# Supplementary material for: Preinjury, injury and post-injury factors leading to death in children and young people who were victims of knife crime in England between 2019 and 2024: a review of the National Child Mortality Database
Source: Emerg Med J. 2026 Apr 20;43(8):e215154. doi: 10.1136/emermed-2025-215154 (PMC13479297; doi:10.1136/emermed-2025-215154)
Supplement: Supplementary file 6 [file emermed-43-8-s007.pdf]

Online supplement 7 Cause of death directly leading to death, as stated on death certificate in section 1a

Incised or stab wounds to:	%	n
Chest	56.9	33
Neck	12.1	7
Abdomen	3.4	2
Upper Limb	1.7	1
Lower Limb	1.7	1
Multiple wounds	12.1	7
Or		
Multi-organ failures (secondary to stab wounds (multiple))	1.7	1
Hypoxic Brain Injury (secondary to stab wounds) (4 to the chest, 1 trunk, 1 multiple)	10.3	6
